# The Critical Role of the Early Evaluation of Iron and Vitamin B12 Deficiency in Pregnancy

**DOI:** 10.7759/cureus.67592

**Published:** 2024-08-23

**Authors:** Vasiliki Gοugοutsi, Abraham Pouliakis, Tsantes Argyrios, Maria Tolia, Nickolaos-Anargyros Nazos, Periklis Panagopoulos, Styliani Kokoris

**Affiliations:** 1 Department of Hematology, University General Hospital "Attikon" School of Medicine/National and Kapodistrian University of Athens, Athens, GRC; 2 Second Department of Pathology, University General Hospital "Attikon" School of Medicine/National and Kapodistrian University of Athens, Athens, GRC; 3 Department of Radiotherapy, University Hospital of Herakleion/University of Crete, School of Medicine, Herakleion/Crete, GRC; 4 School of Nursing, University of Thrace, Alexandroupolis, GRC; 5 Third Department of Obstetrics and Gynecology, University General Hospital "Attikon" School of Medicine/National and Kapodistrian University of Athens, Athens, GRC

**Keywords:** preventive medicine, pregnancy outcomes, vitamin b12 deficiency, iron deficiency, anemia

## Abstract

Background and objective

Anemia is a common hematological disorder during pregnancy, with iron deficiency (ID) being the most prevalent cause globally. It severely affects maternal and fetal health. This study aimed to investigate the prevalence of anemia and its association with iron and vitamin B12 deficiency during pregnancy.

Materials and methods

The study sample consisted of pregnant women attending the 3^rd^ Clinic of Obstetrics and Gynecology, University General Hospital "Attikon", Athens, Greece, with a total of 145 women eventually analyzed. Blood samples were collected from pregnant women during the first, second, and third trimesters; hematological indices, including hemoglobin (HGB), hematocrit (HCT), mean corpuscular volume (MCV), mean corpuscular hemoglobin (MCH), mean corpuscular hemoglobin concentration (MCHC), red blood cell distribution width (RDW), ferritin, and vitamin B12, were recorded. Iron deficiency anemia was defined as HGB <11.0 g/dl in the first trimester and <10.5 g/dl in the second and third trimesters.

Results

Iron deficiency anemia is elevated in the course of pregnancy. A significant proportion of pregnant women had vitamin B12 deficiency during pregnancy, with the prevalence increasing from the first to the third trimester. The study also found that iron supplementation improved hematological indices; especially, pregnant women receiving divalent iron had significantly higher levels of HCT, HGB, and ferritin compared to those receiving trivalent iron.

Conclusions

Screening for iron deficiency anemia should be performed in all pregnant women, and appropriate oral iron therapy should be given as first-line treatment. Early recognition and management of low maternal iron levels are crucial and lead to improved maternal, fetal, and neonatal outcomes. Furthermore, unified international thresholds for ID are required for accurate assessments and appropriate iron supplementing. This study also recommends the screening of vitamin B12 levels as part of the systematic follow-up of pregnant women to identify potential deficiencies and provide appropriate supplementation. Further in-depth studies, particularly related to vitamin B12, are required to provide definitive conclusions and guidance.

## Introduction

Anemia is the most common hematological disorder during pregnancy. The primary causes of anemia are iron deficiency (ID) and vitamin B12 deficiency, particularly in the least developed and developing countries. Globally, over 40% of pregnant women suffer from anemia due to various causes [1]. Generally, the prevalence of anemia during pregnancy has been reported to be highly variable, ranging from 17% to 31% in Europe and North America, 44-53% in Southeast Asia, and 53-61% in Africa [2,3]. The major cause of anemia during pregnancy is ID. Of note, 20% of women worldwide experience ID during their reproductive years [4]. An extensive literature review highlights that ID is a widespread nutritional issue, affecting up to 52% of pregnant women globally [5]. In Europe, iron deficiency anemia affects over 20% of all pregnancies [6]. In Australia, nearly 20% of women begin pregnancy with ID [7]. In the United States, the prevalence of ID among women of childbearing age is reported to be 12%, with higher rates observed in Black and Hispanic women (19% and 22%, respectively).

Iron plays a crucial role during pregnancy and infancy, contributing significantly to hematopoiesis, oxygen transport by red blood cells (RBCs), and overall growth and development. Proper regulation of iron homeostasis is vital for both the mother and the developing fetus, as both the deficiency and excess of iron can disrupt cellular functions [8]. Depending on the severity and duration of anemia and the stage of gestation, there could be different adverse events. The functional consequences of iron deficiency anemia are serious and substantial, leading to poor pregnancy outcomes such as low birth weight, preterm birth, and neurodevelopmental impairment in infants [9]. To meet the accelerating physiologic iron requirements in pregnancy, both dietary iron absorption and the mobilization of iron from stores, the circulating iron should be increased.

Iron supplementation has been recommended during pregnancy to replete its levels and decrease potential risks of ID. Oral iron is usually recommended as first-line therapy, although the most recent intravenous iron interventions seem to replenish iron stores safely and effectively [10]. The current strategy to identify iron deficiency anemia involves assessing various hematological markers, including hemoglobin (HGB), mean corpuscular volume (MCV), red blood cell distribution width (RDW), mean corpuscular hemoglobin concentration (MCHC), and ferritin levels. In many developed countries, it is routine practice to advise all pregnant women to take iron supplements during pregnancy. This approach could be considered effective as it significantly reduces the incidence of anemia, and, by ensuring adequate iron levels, it promotes positive pregnancy outcomes and plays a crucial role in reducing maternal morbidity and mortality [2,11].

Vitamin B12 deficiency during pregnancy is much less common than folate deficiency. However, factors such as a deficient diet (e.g., vegetarian nutrition), individual genetic variations, certain medical conditions, and malnutrition in developing countries make it a significant issue, even though a large amount of cobalamin is stored in the liver [12,13]. Vitamin B12 deficiency (<148 pmol/L) is associated with adverse maternal and neonatal outcomes, including developmental anomalies, spontaneous abortions, preeclampsia, and low birth weight(<2500 g) [2,4]. In addition, vitamin B12 insufficiency (<200 pmol/L) can impair infant growth, psychomotor function, and brain development. Furthermore, B12 gradual depletion is associated with a significant risk of marginal deficiency.

Notably, approximately 20% of pregnant women with lower B12 concentrations have neither metabolic nor clinical signs of B12 deficiency. The primary concern in pregnancy is both maternal and perinatal morbidity and mortality and they remain major challenges in the delivery of safe maternity care worldwide [14]. Our objective in this study was to analyze the prevalence of anemia and its association with iron and vitamin B12 deficiency during pregnancy.

## Materials and methods

Samples

The blood samples to be analyzed were monitored by the 3^rd^ Clinic of Obstetrics and Gynecology of the University General Hospital «Attikon». The study protocol was approved by the Bioethics Committee of the National Kapodistrian University of Athens (12/10/2016). In addition, the study is conformant to the provisions of the World Medical Organization (52^nd^ WMA General Assembly, Edinburgh, Scotland, 2000). Participants' details are considered strictly confidential. The participants were informed about the purposes of this study and the confidentiality of their information, and they agreed to take part voluntarily in this study and signed an informed consent form. The results of red cell indices (HGB, HCT, MCV, MCH, MCHC, RDW, ferritin, and, where necessary, vitamin B12 were recorded. Measurements were performed during the first trimester (12th week), the second (22nd-23rd week), and the third trimester (32nd-33rd week) of pregnancy. The same patients underwent these investigations in all three trimesters.

The blood samples for red cell indices were collected and processed by the routine procedures in the laboratory, using, approximately 3 ml of a venous blood sample, which was collected by a trace element-free syringe and immediately transferred into EDTA (ethylenediaminetetraacetic acid) element-free heparin vials. The EDTA blood was analyzed on the same day using the Counter Sysmex XE-2100 (ROCHE, Basel, Switzerland) system. Furthermore, a venous blood specimen of 5 ml was collected for serum ferritin and vitamin B12 into a vacuum tube: WEGO 5 ml (WEGO, Weihai, China). These samples were analyzed using an automated Roche Modular E170 Analyzer.

Standard operating procedures were strictly followed, to maintain the quality of the laboratory results. The results of hematological indices were recorded in Excel spreadsheets where data such as age, number of pregnancies, abortions, last menstrual period, chronic diseases (e.g., malabsorption syndrome that affects the absorption of iron), and the values of the hematological biomarkers found, were recorded. Before the first blood sampling during the initial visit, we collected information to evaluate and identify the physical profile of pregnant women. Demographic information was gathered for ethnicity, age, menstrual, obstetric, and medical history (especially hemoglobinopathies); the primary measured outcome was the iron levels of women and the secondary outcome was the vitamin B12 levels.

According to UK guidelines on the management of iron deficiency in pregnancy, anemia was defined in our study as HGB level <11.0 g/dl in the first trimester and <10.5 g/dl in the second and third trimesters.

Statistical analysis

The chi-square test was employed for categorical variables and the Mann-Whitney U test or the Students t-test were used to compare continuous variables, depending on normality conditions. Furthermore, we applied general linear models to assess the association between iron supplements and B12 deficiency. We examined the interaction between iron supplements and maternal HGB concentration by adding a product term to the regression model. Pearson’s correlation analysis between birth weight and HGB concentration was carried out among women with high and very high baseline HGB concentrations in each study group. P-values were compared with a significance level of α=0.05. Statistical analyses were performed by using SPSS Statistics v. 22.0 (IBM Corp., Armonk, NY) and within the environment of SAS for Windows (version 9.4). All tests were two-sided.

## Results

**Figure 1:**
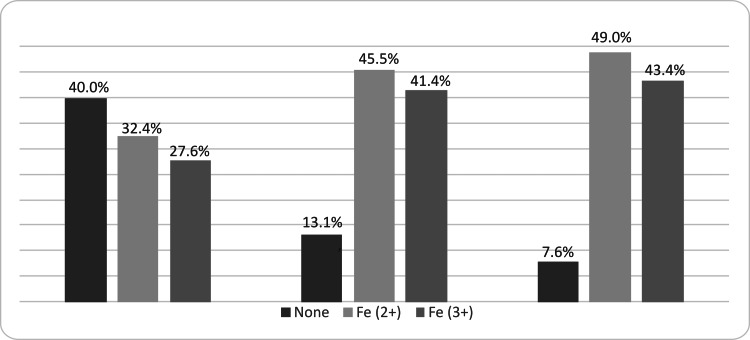
Proportion of participants who received iron supplements per trimester

A total of 145 pregnant women (mean age: 31.9 ±5.5 years; range: 15-46 years) were enrolled in the study. Participants had a mean of 1.9 ±1.1 pregnancies (range: 0-9) and 0.14 ±0.43 miscarriages (range: 0-2). Figure 1 illustrates the proportion of participants who received iron supplements per trimester.

**Figure 2:**
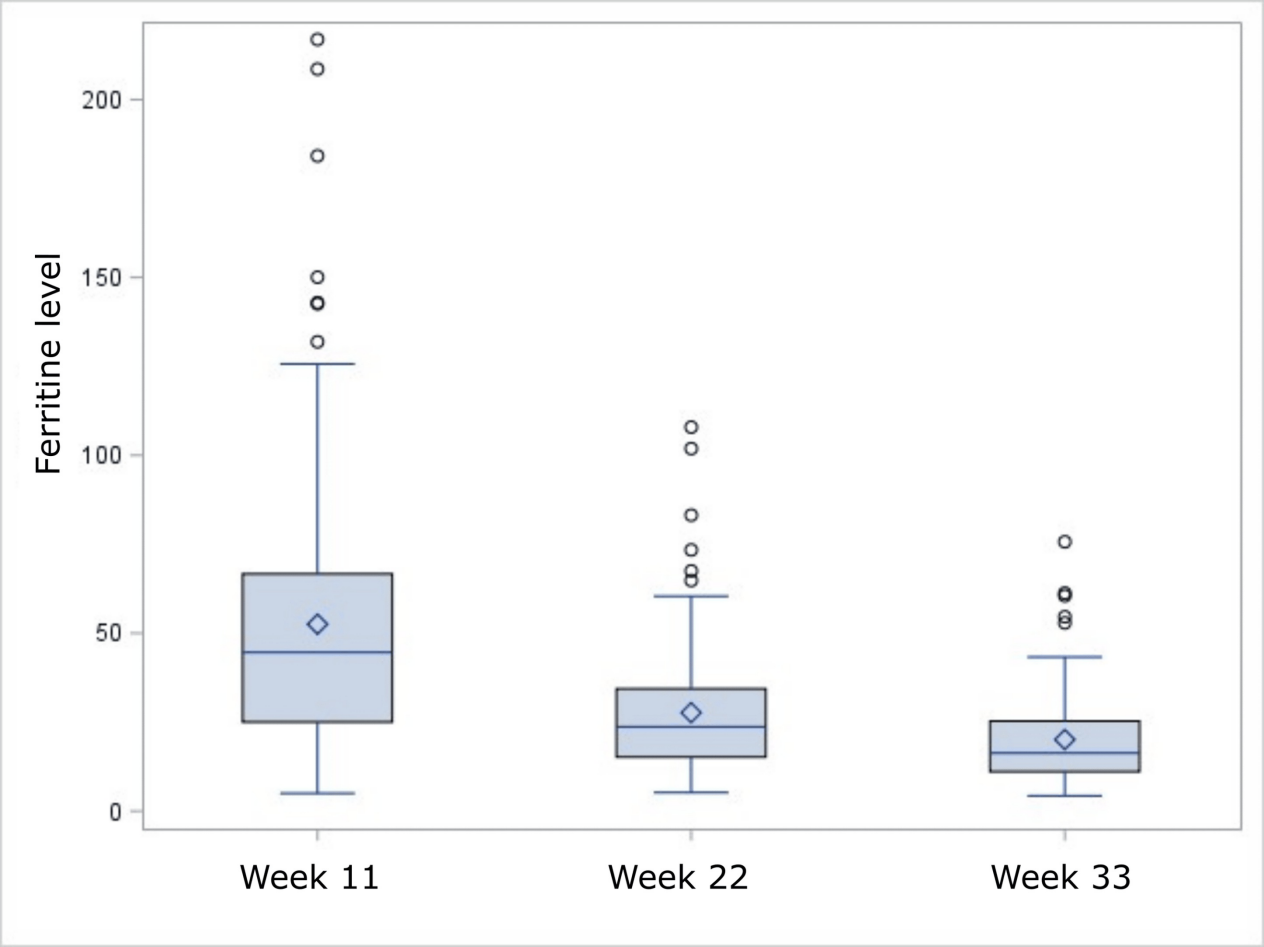
Decrease in ferritin levels during three trimesters of pregnancy (p<0.001) In each diagram, the box limits indicate the lower (first) and higher (third) quartiles; horizontal lines within the boxes indicate the median value, while the limits of the whiskers indicate minimum and maximum values after excluding outliers. Mean values are presented as diamond symbols and circles outside the whisker areas indicate outliers. P-value <0.05 is considered significant

The hematological variables in the cohort are shown in Table 1. As the gestation period progressed, the number of women with normal (or even above normal) levels of ferritin decreased, until the third trimester, when 84% (N=122) of women had ferritin <30 ng/mL (Figure 2).

**Table 1:** Hematological variables in the study groups at enrollment (first trimester) and second and third trimesters of pregnancy HCT: hematocrit; HGB: hemoglobin; MCH: mean corpuscular hemoglobin; MCHC: mean corpuscular hemoglobin concentration; MCV: mean corpuscular volume; PLT: platelets; RBC: red blood cells; RDW: red cell distribution width; SD: standard deviation; WBC: white blood cells

Variables	First trimester	Second trimester	Third trimester
RBC (cells x 10^6^) (mean ±SD)	4.30 ±0.43	6.72 ±34.06	3.89 ±0.40
WBC (κ/μl) (mean ±SD)	8.59 ±2.21	10.08 ±2.41	10.26 ±2.72
HCT (%), normal levels (%) (mean ±SD, % of normal)	37.47 ±2.94, 93.8%	34.41 ±2.82, 69.7%	33.94 ±3, 61.4%
HGB (g/dl), normal levels (%) (mean ±SD, % of normal)	12.35 ±0.93, 90.3%	11.44 ±0.94, 67.6%	11.43 ±1.04, 67.6%
RDW (%) (mean ±SD)	14.18 ±1.83	13.90 ±1.62	14.12 ±2.99
MCV (fl), normal levels (%) (mean ±SD, % of normal)	88.83 ±9.66, 89%	88.47 ±8.67, 87.6%	86.90 ±8.16, 86.2%
MCH (pg) (mean ±SD)	28.83 ±3.07	30.80 ±17.02	29.40 ±3.26
MCHC (g/dl) (mean ±SD)	32.47 ±1.89	34.46 ±14.69	33.48 ±3.02
PLT (K/μl) (mean ±SD)	247.38 ±62.09	235.86 ±59.40	228.39 ±56.60
Ferritin (μg/ml) (mean ±SD)	52.52 ±37.58	27.64 ±17.79	20.08 ±12.35
B12 (pg/ml) (mean ±SD)	319.56 ±135.07	268.47 ±94.12	231.99 ±85.16
Iron deficiency anemia (%)	4.1	11.7	24.1
Iron deficiency (%) (none, mild, moderate)	68.2, 22.1, 9.7	35.2, 40.0, 24.8	16.6, 40.0, 43.4

Women who received iron supplements in the first trimester had statistically significantly higher ferritin levels in the second trimester compared to those who did not receive iron in the first trimester (31.11 ±17.78 vs. 22.45 ±16.63 ng/ml, p=0.0037). Women who received iron in the second trimester had significantly lower MCHC levels in the second trimester than those who did not receive iron in the second trimester (33.19 ±1.67 vs. 42.86 ±40.27 g/dl, p=0.007). Pregnant women who received iron in the third trimester had significantly elevated HCT levels in the third trimester compared to those who did not receive it (34.10 ±2.87 vs. 32.31 ±3.89, p=0.007), indicating that iron supplementation fulfills the increasingly higher needs of the woman’s body as pregnancy proceeds.

Type of iron (divalent vs. trivalent)

Women who received divalent iron supplements from the first trimester of pregnancy had significantly higher levels of HCT and HGB in the second trimester and ferritin, HCT, and HGB in the third trimester than those who received trivalent iron supplements (Table 2).

**Table 2:** Comparison between participants who received divalent iron vs. those who received trivalent iron per trimester ^+^Mann Whitney U test; ^*^t-test P-value <0.05 is considered significant HCT: hematocrit; HGB: hemoglobin; MCH: mean corpuscular hemoglobin; MCHC: mean corpuscular hemoglobin concentration; MCV: mean corpuscular volume; PLT: platelets; RBC: red blood cells; RDW: red cell distribution width; SD: standard deviation

Variables	Divalent iron - first trimester (n=47), mean ±SD	Trivalent iron - first trimester (n=40), mean ±SD	P-value
MCH (pg) first trimester	29.54 ±2.19	28.34 ±3.29	0.045^+^
MCHC (g/dL) first trimester	32.71 ±1.45	31.77 ±2.06	0.014^+^
	Divalent iron - second trimester (n=66), mean ±SD	Trivalent iron - second trimester (n=60), mean ±SD	P-value
MCH (pg) first trimester	29.31 ±2.48	28.13 ±3.65	0.034^+^
HCT (%) second trimester	34.98 ±2.73	33.74 ±2.81	0.013^+^
HGB (g/dL) second trimester	11.59 ±0.87	11.19 ±0.93	0.014^*^
MCV (fL) second trimester	90.68 ±7.79	85.95 ±9.48	0.003^+^
	Divalent iron - third trimester (n=70), mean ±SD	Trivalent iron - third trimester (n=62), mean ±SD	P-value
MCH (pg) first trimester	29.39 ±2.43	28.12 ±3.60	0.017^+^
HCT (%) second trimester	35.10 ±2.90	33.66 ±2.63	0.003^+^
HGB (g/dL) second trimester	11.66 ±0.93	11.17 ±0.89	0.003^*^
MCV (fL) second trimester	90.80 ±7.70	85.73 ±9.11	0.001^+^
RDW (%) second trimester	13.67 ±1.46	14.26 ±1.84	0.042^+^
Ferritin (ng/mL) third trimester	22.20 ±11.59	17.17 ±12.77	0.019^+^
HCT (%) third trimester	34.88 ±2.82	33.22 ±2.69	0.001^*^
HGB (g/dL) third trimester	11.76 ±0.94	11.09 ±1.08	0.001^*^
MCH (pg) third trimester	30.28 ±2.43	28.42 ±3.82	0.001^+^
MCV (fL) third trimester	89.00 ±7.07	84.68 ±8.93	0.002^+^

Vitamin B12 (normal vs. low levels)

No statistically significant differences were found between participants who had normal levels of vitamin B12 compared to those with low levels. Participants who had normal vitamin B12 levels in the second trimester had significantly higher levels of vitamin B12, MCHC, and MCH, and lower levels of PLT, RBC, and RDW in the first trimester and significantly higher levels of MCH and MCV in the second trimester than participants with low levels of vitamin B12. Moreover, participants who had normal vitamin B12 levels in the third trimester had significantly higher levels of vitamin Β12 and MCHC in the first trimester, vitamin Β12 in the second trimester, and HGB, MCH, and MCV in the third trimester (Table 3).

**Table 3:** Comparison between participants with normal vs. low levels of vitamin B12 in the second and third trimesters of pregnancy All statistics performed with the Mann-Whitney U test. P-value <0.05 is considered significant

Variables	Normal levels - second trimester (n=85), mean ±SD	Low levels - second trimester (n=16), mean ±SD	P-value
Β12 (pg/mL) first trimester	355.50 ±127.09	212.79 ±70375	<0.001
MCHC (g/dL) first trimester	32.82 ±1.71	32.07 ±1.86	0.046
MCH (pg) first trimester	29.36 ±2.48	27.78 ±3.70	0.010
PLT (K/μl) first trimester	244.76 ±58.03	272.47 ±74.03	0.039
RBC (10/L) first trimester	4.25 ±0.37	4.45 ±0.47	0.023
RDW (%) firsttrimester	13.79 ±1.61	14.66 ±1.94	0.017
MCH (pg) second trimester	29.99 ±2.65	28.37 ±3.67	0.011
MCV (fL) second trimester	90.17 ±8.21	85.04 ±8.29	0.004
	Normal levels - thirdtrimester (n=79), mean ±SD	Low levels - third trimester (n=58), mean ±SD	P-value
Β12 (pg/mL) first trimester	367.46 ±142.29	240.60 ±79.36	<0.001
MCHC (g/dL) first trimester	32.73 ±1.89	32.06 ±1.86	0.043
Β12 (pg/mL) second trimester	316.12 ±84.67	199.81 ±55.83	<0.001
HGB (g/dL) 3^rd^ trimester	11.59 ±1.05	11.24 ±0.99	0.049
MCH (pg) 3^rd^trimester	29.99 ±2.77	28.74 ±3.73	0.025
MCV (fL) 3^rd^ trimester	88.33 ±7.60	85.57 ±8.53	0.049

Iron doses in all three trimesters were correlated with ferritin. The tests were performed using Spearman's correlation coefficient. The results for each correlation are presented in Table 4. In many cases, a statistically significant correlation was found (p<0.05), but the degree of correlation was moderate to low.

**Table 4:** Correlation of iron dose with ferritin levels per trimester of pregnancy Spearman's correlation coefficient and the relevant p-value are presented in each cell. P-value <0.05 is considered significant

	Iron dose - first trimester	Iron dose - second trimester	Iron dose - third trimester
Ferritin - first trimester	0.1654, p=0.0484	0.1227, p=0.1442	0.1397, p=0.0962
Ferritin - second trimester	0.3376, p<0.0001	0.2599, p=0.0017	0.2538, p=0.0022
Ferritin - third trimester	0.1698, p=0.0427	0.2020, p=0.0156	0.2224, p=0.0076

Table 5 presents the results of linear regression with the dependent variable iron deficiency anemia in the second and third trimesters of pregnancy. The analysis shows that iron deficiency anemia in the second trimester was predicted by ferritin levels in the first trimester (p<0.05), ferritin levels in the second trimester (p<0.05), iron deficiency anemia in the second trimester (p<0.05) and iron dose in the first trimester (p<0.05). Also, iron deficiency anemia in the third trimester was predicted by ferritin levels in the first trimester (p<0.05), ferritin levels in the second trimester (p<0.05), iron deficiency anemia in the second (p<0.05), iron deficiency anemia in the third trimester (p<0.05), and divalent iron in the third trimester (p<0.05).

**Table 5:** Linear regression results with dependent variable iron deficiency in the second and third trimesters of pregnancy P-value <0.05 is considered significant CI: confidence interval

Variables	β (95% CI)	P-value
Iron deficiency anemia - second trimester		
Ferritin (ng/mL) first trimester	1.5 (1.3-1.7)	0.001
Iron deficiency anemia second trimester	1.25 (0.14-1.59)	0.001
Ferritin (ng/mL) second trimester	29.4 (25.6-33.3)	0.001
Iron dose (mg) first trimester	250 (200-500)	0.001
Iron deficiency anemia - third trimester		
Ferritin (ng/mL) first trimester	125 (91-200)	0.001
Ferritin (ng/mL) second trimester	43.5 (34.5-55.6)	0.001
Iron deficiency anemia second trimester	1.4 (1.1-2.9)	0.001
Iron deficiency anemia third trimester	1.2 (1.1-1.6)	0.001
Iron type third trimester, divalent	-4.0 (2.0-125)	0.043

## Discussion

In a cross-sectional study involving 253 anemic and 147 non-anemic pregnant women, hematological parameters such as ferritin, iron, total iron binding capacity (TIBC), transferrin saturation (TfS), C-reactive protein (CRP) and bilirubin were measured. The results showed that anemic women had significantly lower levels of HGB, HCT, MCV, iron, ferritin, and transferrin saturation and higher levels of WBC, RDW, TIBC, and positivity of CRP compared to non-anemic pregnant women, suggesting that anemia in pregnancy is associated with a significant disorder in hematological and iron indices due to ID [16]. In another study of 231 healthy Japanese women without anemia who did not receive iron supplementation before the second-trimester blood test, the hematological parameters were assessed during the first, second, and third trimesters. This study showed that 20% of women developed anemia in the third trimester while the first-trimester RBC, HGB, HCT, and ferritin levels were significantly lower in women with anemia in the third trimester compared to those who did not have anemia. Also, first-trimester HGB levels were significantly better predictors of anemia during the third trimester than the indices of iron storage, including serum iron, ferritin, and TIBC levels [17].

Furthermore, in a study of 182 patients who entered prenatal care, a practice protocol was applied to correlate the serum ferritin levels with the proportion of women with anemia and ID. The results showed that ID was observed in 54% of patients with anemia (12 mg/dL ferritin was the cutoff for ID) suggesting that screening for anemia in pregnancy should be further evaluated [18]. Finally, in another study, HGB and ferritin concentrations were measured in 170 pregnant women at term to determine the prevalence of anemia. The results showed that the mean levels of HGB and ferritin were 10.9 ±1.9 and 47.84 ±98.39 µg/L respectively, while anemia at term was observed in 46.4% of women who received 200 mg elemental iron in three divided doses and 5 mg folic acid daily beginning from the second trimester. The study revealed that 43.5% of the pregnant women at term were mild to moderately anemic, while 2.9% had severe anemia (HGB <7 g/dL) [19].

In the present study, prenatal supplementation of iron was associated with improved hematological indices. Of note, pregnant women who had iron deficiency anemia at week 33 had significantly lower ferritin levels (p<0.05) in all three measurement periods. They also had significantly lower levels of HCT (p<0.05), HGB (p<0.05), and MCV (p<0.05) at week 33 of gestation. Our results align with the study by Noshiro et al. [17] who reported that women with anemia in the third trimester had significantly lower levels of RBC, HGB, HCT, and ferritin in the first trimester compared to those without anemia. Also, we noticed that the iron supplements taken by pregnant women greatly influenced the outcome of anemia and the improvement of hematological indices. Interestingly, in the study by Adediran et al. [20], anemia at term was seen only in 46.4% of women who received elemental iron and folic acid at the beginning of the second trimester.

The number of women receiving iron supplementation increased gradually. In the first trimester, 40% (n=58) were not receiving any treatment; in the second trimester, the rate was 13.1% (n=19), while only 7.7% (n=11) received no iron treatment in the third trimester. Given that a significant number of women did not receive iron treatment only in the first trimester of pregnancy (40%), it is easier to identify statistically significant differences for this trimester compared to the other two, where the use of iron supplements gradually increases, resulting in a much smaller sample size for these groups.

Pregnant women who received divalent iron from the first trimester had significantly higher levels of HCT and HGB in the second trimester and Ferritin, HCT, and HGB in the third trimester than those who received trivalent iron. Similarly, the study by Berber et al. [21] showed that in women with iron deficiency anemia treated with oral ferrous glycine sulfate tablets, the increase of the mean HGB and HCT was significantly higher compared to those who treated with oral ferric protein succinylate tablets. In addition, the mean serum vitamin B12 levels at the end of the third trimester of pregnancy (231.0 ±1.04 pg/mL) were statistically significantly less than the mean serum vitamin B12 levels (268.5 ±94.1 pg / mL) in the second trimester. Our results align with the study by Behere et al. [22], which confirmed the high prevalence (50-70%) of B12 deficiency in Western and Southern parts of India while noticing that vitamin B12 measurements were lower in the third trimester of pregnancy as compared to early pregnancy.

Based on World Health Organization recommendations, vitamin B12 deficiency has been defined as a serum vitamin B12 concentration of less <200 pg/mL. As per this limit, 15.1% (n=22) of pregnant women experienced vitamin B12 deficiency at the end of the first trimester (12th week). A significantly higher rate of 26.2% (n=38) of pregnant women experienced vitamin B12 deficiency at the end of the second trimester (23rd-24th week). Subsequently, the percentage of pregnant women who had vitamin B12 deficiency in the 33rd week of gestation was almost 42.1% (n=61), almost close to half of the women in the study sample. To sum up, in our measurements, we noticed that vitamin B12 levels are significantly reduced at a remarkable rate in pregnant women, with the progress of pregnancy from the first to the third trimester. Other studies have also made such observations [22].

Screening for iron deficiency anemia is recommended for all pregnant women, and appropriate oral iron therapy should be initiated as first-line treatment [23]. Early recognition and management of low maternal iron status is crucial and associated with improved maternal, fetal, and neonatal outcomes. Nevertheless, existing international guidelines for the testing and management of maternal iron deficiency anemia are variable, and, as a consequence, some high-risk women are taking inadequate doses of iron to prevent anemia [24,25].

Our study has certain limitations, primarily its low sample size; of the 325 women initially enrolled in the study, only 145 were monitored for the complete study duration or had valid data (i.e., 44.6%). The second limitation is related to the study design, which was not especially equipped to monitor B12 but instead other iron-related markers. Thus, larger studies specifically designed for B12 monitoring (i.e., randomized controlled trials with two arms; one with B12 supplement and one without) could help us gain deeper insights into the role of B12 supplementation during pregnancy. A third limitation is related to factors that may contribute to anemia but were not recorded during this study, including the socioeconomic status of women and their families as well as their dietary habits (vegetarian or nonvegetarian).

## Conclusions

This study found that ID progresses throughout the course of pregnancy. All RBC markers or biomarkers related to iron used in this survey were notably lower as pregnancy advanced from the first to the third trimester, with particularly high deficiency documented by the 33rd week. Measurement of red cell indices aids and facilitates the diagnosis of ID, and hence we recommend screening for all pregnant women by obstetricians. Furthermore, ID can persist despite supplementation. The absence of established guidelines necessitates restructuring, and unified international thresholds for ID are needed to ensure accurate assessments and appropriate iron supplementation. It is also noteworthy that in a significant number of pregnant women, vitamin B12 levels gradually decrease. Our study indicates that B12 insufficiency during pregnancy is common and vitamin B12 concentrations decrease from the first to the third trimester. While folic acid supplementation during pregnancy is widely recommended, vitamin B12 supplementation has received less attention.

Overall, it is prudent and beneficial to consider additional blood indices, biomarkers, and measurements in pregnant women to accurately diagnose iron deficiency. We recommend including screening for vitamin B12 levels as part of systematic follow-up during pregnancy, which could prove valuable. Further comprehensive studies, particularly focusing on vitamin B12, are necessary to draw definitive conclusions, provide guidance, and establish the benefits of supplementation.
